# Maternal diet before and during pregnancy and risk of asthma and allergic rhinitis in children

**DOI:** 10.1186/s13223-019-0353-2

**Published:** 2019-06-22

**Authors:** Nour Baïz, Jocelyne Just, Julie Chastang, Anne Forhan, Blandine de Lauzon-Guillain, Anne-Marie Magnier, Isabella Annesi-Maesano, the EDEN Mother-Child Cohort Study Group

**Affiliations:** 1 Epidemiology of Allergic and Respiratory Diseases Department (EPAR), Pierre Louis Institute of Epidemiology and Public Health (IPLESP UMRS 1136), Saint-Antoine Medical School, Sorbonne Université and INSERM, 27 Rue Chaligny, 75571 Paris Cedex 12, France; 2 Département d’Allergologie, Hôpital Trousseau AP-HP–UPMC Paris 6, Paris, France; 3 Department of General Practice, Medical School Saint Antoine, Sorbonne Université, 75012 Paris, France; 4 INSERM, UMR1153 Center for Research in Epidemiology and StatisticS (CRESS), Research Team on Early Life Origins of Heath (EAROH), Paris, France; 5 Paris Descartes University, Paris, France; 6 INRA, U1125 Center for Research in Epidemiology and StatisticS (CRESS), Research Team on Early Life Origins of Heath (EAROH), Paris, France

**Keywords:** Diet, Preconception, Pregnancy, Allergic diseases, Wheezing, Allergic rhinitis, Atopic dermatitis, Mother–child cohort

## Abstract

**Background:**

Consumption of certain foods during pregnancy has been shown to have beneficial effects on childhood asthma and allergic disease development and aggravation. However, most studies provide conflicting results and the relationships between maternal preconceptional diet and risks of childhood asthma and allergic disease have not previously been explored. The objective of this study was to assess maternal diet during the year before pregnancy and the last 3 months of pregnancy and investigate their associations with the risks of asthma, wheezing, allergic rhinitis and atopic dermatitis in young children.

**Methods:**

The study sample consisted of 1140 mother–child pairs from the EDEN cohort. Mothers had responded to the food frequency questionnaires used to assess diet before and during pregnancy. Children were followed up using health questionnaires. The health outcomes studied were: asthma, wheezing, allergic rhinitis and atopic dermatitis by the age of 3 years.

**Results:**

Using multivariable-adjusted logistic regression models, significant inverse associations were observed between cooked green vegetable consumption before pregnancy and childhood asthma; consumption of eggs and raw vegetables before and during pregnancy, consumption of grains before pregnancy, and consumption of cooked green vegetables during pregnancy and allergic rhinitis. For the first time, a significant positive association was found between meat intake during the preconceptional period and a risk of wheezing, allergic rhinitis and atopic dermatitis.

**Conclusions:**

Based on our findings, preconceptional and prenatal maternal intake of certain type of food groups may be preventive against asthma, wheezing and allergic rhinitis, whereas higher maternal intake of meat before pregnancy may increase the risk of wheezing, allergic rhinitis and atopic dermatitis in young children.

## Introduction

The prevalence of allergies and asthma is increasing, suggesting the influence of the environment [1, 2]. Maternal diet during pregnancy is often considered a possible factor in the development of atopy in children [3]. However, whether gestational diet contributes to protecting their children from asthma and allergic dieases has been poorly investigated and the preconceptional period has not previously been explored. The answer to this question is important because diet is modifiable and it may thus be possible to carry out related public health interventions to prevent the development of asthma and allergic diseases during childhood.

Because prenatal life is a crucial period in immune system development, a number of studies have examined the role of intrauterine exposures in the etiology of allergic diseases [4]. Pregnancy is a physiological situation involving high anabolic activity, requiring sufficient intake of micronutrients, trace elements and vitamins [5]. During gestation, essential nutrients are transferred from the mother into the fetal circulation through the placenta [6]. Consequently, dietary factors associated to allergic diseases likely begin to exercise their influence in utero. In this context, it has been suggested that maternal diet during pregnancy may influence fetal immune responses and thus be involved in predisposition to allergies in childhood [7, 8].

Foods consist of a mixture of nutrients whose combined effects could be greater than the sum of their individual effects, whether or not they protect against asthma and allergic diseases. The consumption of certain foods and food groups by pregnant women has been shown in various studies to have beneficial effects on asthma and allergic diseases [9–12]. However, the relationships between maternal diet before the beginning of pregnancy and risks of childhood asthma and allergic disease have not previously been explored.

It is thus of interest to investigate the relationships of groups of similar foods consumed by the mothers before and during pregnancy to the risks of childhood asthma and allergic diseases. The objective of the present study was to assess maternal dietary intake by food group during the year before and during the last 3 months of pregnancy and to explore the associations between maternal intake of certain categories of foods and the risks of asthma, wheezing, allergic rhinitis and atopic dermatitis in children from the EDEN birth cohort followed-up to the age of 3 years.

## Materials and methods

### Population

The required data were available for 1140 of the 2002 women included in the EDEN study [13]. Mother–child pairs were recruited in the EDEN Prospective Birth Cohort Study (http://eden.vjf.inserm.fr). The primary aim of the EDEN cohort is to identify prenatal and early postnatal nutritional, environmental, and social determinants associated with children’s health and normal and pathologic development. Pregnant women seen for a prenatal visit at the departments of Obstetrics and Gynecology of the University Hospital of Nancy and Poitiers before their 24th week of amenorrhea were invited to participate. Enrollment started in February 2003 in Poitiers and September 2003 in Nancy; it lasted 27 months in each center. Women with speaking and writing abilities in French who did not have type 2 diabetes diagnosed before pregnancy and did not plan to deliver outside the university hospital or move out of the region within 3 years were included in the study. Multiple pregnancies were excluded. Among eligible women, 55% (2002 women) agreed to participate (1034 women in Nancy and 968 in Poitiers). Of the 2002 mother–child pairs included in the EDEN study, 1140 of them had complete data by the child’s age of 3 years. The missing data relate mainly to women who decided to withdraw from the study, women who were lost to follow-up, and miscarriages or fetal deaths. Analyses were performed on results from the 1140 women who responded to the two food frequency questionnaires (FFQs) and completed annual health questionnaires up to age 3.

### Ethics statement

The ethical committees who approved the study are: Comité Consultatif pour la Protection des Personnes dans la Recherche Biomédicale, Le Kremlin-Bicêtre University hospital, and Commission Nationale de l’Informatique et des Libertés. The study was approved on 12 December 2002. Written consent was obtained from the mother for herself at the beginning of the study and from both parents for the newborn child after delivery.

### Maternal dietary assessment

The FFQ completed by mothers in the EDEN cohort was very similar to the one developed for the Fleurbaix-Laventie Ville Santé study [14], which was validated using a series of 24-h recalls [15]. The EDEN questionnaire included some additional items about intake of fish and foods rich in folates, n-3 FAs and vitamin A. The FFQ included 137 foods and food groups assessed on a seven-item scale, from “never” to “more than once a day.” The questions concerned both single food items (e.g., lamb chop, lettuce, banana, orange juice) and mixed dishes (e.g., cassoulet, paëlla). Participants had to indicate portion sizes for each food, using a portion-size booklet with photographs of portion sizes for 12 foods and drinks (three sizes each), drawn from a manual used in the SU.VI.MAX (SUpplémentation en Vitamines et Minéraux Anti-oXydants [supplementation with antioxidant vitamins and minerals]) study [16]. The questionnaire also included questions on cooking methods and on fats and oils used in cooking and seasoning. For other foods, the portion consumed was assumed to be a standard portion assessed for the French adult population [17]. To calculate the food intake for each item, the portion consumed (in g) was multiplied by the frequency declared (per day). Some changes were made after a validation step for the questionnaire by comparing it to repeated 24-h recalls. We estimated mean consumption for a food group by adding together estimated mean consumption of all the foods included in it.

Individual total energy intakes were calculated for all women by multiplying the intake (in g per day) by the energy value of each food. Energy values were obtained from the SU.VI.MAX nutrient composition database [18, 19]. The estimated total energy intake of our women’s sample varied between 1000 and 5000 kcal/day.

In our study, we focused on categories of foods known to be rich in nutrients (vitamin E, vitamin C, vitamin A, zinc, …) and PUFA (n-6 FA and n-3 FA) which have a previously demonstrated association with childhood asthma and allergic diseases [20]. We chosed to examine the following categories of foods: eggs, meats, fish, cheeses, milk, cooked green vegetables, raw vegetables, grains and fruits. The foods included in each food group are listed in Table 1.

**Table 1:** Foods included in the food groups used for data analyses

Food group	Foods included
Eggs	Fried eggs, omelettes, poached eggs, hard- and soft-boiled eggs
Meats	“Grilled, roasted, or sautéed” beef, ground beef patty, pork (except charcuterie), veal, lamb, or mutton
Poultry	Poultry (chicken, turkey, …), rabbit
Fish	Fresh or frozen fish (cod, pollack, whiting, sole, trout, …), canned fish in oil (tuna, sardines, …), smoked or salted fish (salmon, herring, …), breaded fish, fish-based ready-made dishes, shellfish (mussels, oysters, shrimp)
Cheeses	Emmental, Gruyère, Comté, Beaufort, Bonbel, Babybel, Gouda, Edam, Cantal, Tommes, Morbier, St. Nectaire, Reblochon, …. Brie, Camembert, Pont l’Evêque, Munster, Vacherin, St Marcelin, Caprice des Dieux-type cheese, … Roquefort, blue cheese of any origin Chèvre, fresh cheese (brands such as Le Tartare, Kiri, …)
Milk	Whole milk, semi-skimmed milk, skimmed milk
Fats	Butter, margarine, non-fat-reduced crème fraîche
Cooked green vegetables	Green beans, endives, spinach, cress, leek, cabbage, cauliflower, Brussels sprouts, broccoli, carrots, zucchini, eggplant (ratatouille, …), peas, other vegetables (turnip, chard, …), vegetable soup, corn, pumpkin/squash, sweet potatoes, soybean sprouts
Raw vegetables	Green salad, endives, cress, spinach, grated carrot, celery, tomatoes, beets, cabbage, cucumber, radish, avocado, sprouts, …
Grains	Bread (white, wholewheat,…), biscotti, crispbreads, cereal
Fruit	Apricots, melon, mangoes, peaches, prunes, cherries, strawberries, raspberries, bananas, kiwi, citrus fruit, apples, pears, grapes, other fresh fruit, dried apricots or peaches, other dried fruits
Fruit juices	Orange juice, grapefruit juice, pineapple juice, apple juice, grape juice, etc.

The mothers completed the first FFQ at the time of recruitment, before the 24th week of amenorrhea, indicating their usual dietary intake during the year before the pregnancy. The second FFQ was completed in the first days after delivery, and concerned their usual intake during the three final months of pregnancy. The questionnaires were completed by mothers with the assistance of the midwives if needed. For our study, we chose to examine maternal food intake during the year before the pregnancy and the three final months of pregnancy, which can be expected to reflect the mother’s diet during pregnancy and which is a period during the mothers are less likely to experience nausea and vomiting and during which the organs and immune system of the fetus is still developing.

### Health variables

At the age of 1, 2 and 3, the parents completed a questionnaire including questions on asthma, wheezing, allergic rhinitis, based on the validated phase I questionnaire from the International Study of Asthma and Allergies in Childhood (ISAAC) [21], and doctor-diagnosed atopic dermatitis. The questionnaires were sent by mail were completed and returned back by the parents within 1 month after the distribution. If needed, parents could ask for assistance to complete the questionnaire by telephoning the midwives. Asthma was defined as parental report of doctor-diagnosis of asthma plus either one or more attacks of wheeze or asthma medication in the last 12 months. Wheeze was defined as present if the parents answered ‘‘yes’’ to the question ‘‘Has your child had wheezing or whistling in the chest in the preceding 12 months?’’. Allergic rhinitis was defined as sneezing, nasal congestion, or rhinitis, other than with respiratory infections, accompanied by eye itching and tearing during the previous 12 months [22]. And atopic dermatitis was defined as atopic dermatitis diagnosed by a doctor. Given the uncertainty of diagnoses of asthma and allergic rhinitis in early childhood, the responses at age of 1, 2 and 3 years were incorporated into calculated lifetime prevalence at age 3. Likewise, the calculated prevalence of wheezing and atopic dermatitis by the age of 3 included the responses from each year up to this age. Asthma, wheezing, allergic rhinitis and atopic dermatitis were thus defined as present at age 3 when parental responses indicated that they had been present in any of the first 3 years.

### Other variables

We collected information on potential confounding factors linked to the children’s health variables, including the newborn’s sex, birth weight, gestational age, birth season, number of siblings at birth (0, 1–2, ≥ 3), exclusive breastfeeding for 4 or more months, mother’s age at the time of the child’s birth (< 25 years, 25–34 years, or > 34 years), body-mass index (BMI) before the start of the pregnancy (18.5–24.9, 25.0–29.9, 30.0–34.9, or 35.0–39.9 kg/m^2^), maternal and paternal allergic history (based on a medical diagnosis of allergic diseases, including asthma, allergic rhinitis, atopic dermatitis, or food allergies), mother’s and father’s level of education (primary or below, secondary, university or above), household income (≤ 3000 euros vs. > 3000 euros per month, the median income of the study population), city of home (Nancy or Poitiers), tobacco smoking during the pregnancy, tobacco smoking before the pregnancy, the child’s exposure to environmental tobacco smoke between the ages of 0 and 3 years, humidity in the home (0 to 3 years), and supplement intake (vitamins [excluding vitamin D] and minerals) before and/or during the pregnancy (yes/no).

### Statistical analysis

We determined the characteristics of our study population (*n* = 1140) and of the entire cohort (*n* = 2002) by calculating the frequencies of categorical variables and the means and standard deviations of continuous variables. The characteristics of our population were compared to those of the entire cohort using Chi squared tests on the frequencies of categorical variables, and Mann–Whitney U tests on the means of continuous variables.

Mean consumption of foods in each food group was categorized into three tertile groups, consisting of individuals with low, moderate, and high intake of the foods in the food group. In the regression models, moderate and high intake were compared to low intake (which was used as the reference).

We analyzed the associations between the health variables and maternal food consumption by consumption level using multiple logistic regression models. We estimated odds ratios (OR) and 95% confidence intervals (95% CI) for each health variable.

Bivariate analyses were performed on each pairing of a health variable with a potential confounding factor. In a first phase, all variables whose association to a health event had a *P* value < 0.30 were identified. In the second phase, those involving an OR difference of at least 20% were selected and included in the multivariate models. In addition to the confounding factors included in the models on the basis of a statistically significant association with a health variable, we also selected adjustment variables based on a known relationship to asthma and allergic diseases, independently of their association with maternal food intake or their statistical association with health variables. These included: the sex of the newborn [23], maternal BMI before the pregnancy [24], birth weight [25], birth season [26], number of older siblings [27], exclusive breastfeeding [28] and daily energy intake during the pregnancy [29].

The logistic regression models were adjusted for the following factors: maternal age, maternal BMI before pregnancy, active smoking before and during pregnancy, the child’s exposure to environmental tobacco smoke (between the ages of 0 and 3 years), humidity in the home (0–3 years), maternal atopy, the child’s sex, birth weight, birth season, exclusive breastfeeding ≥ 4 months, number of older siblings, mother’s education, household income, supplementation before and during pregnancy and total daily energy intake.

Finally, to study the modulation of the effect of maternal food intake on child health variables by maternal atopy, we stratified the population by maternal history of asthma and allergies and tested the interaction terms (maternal food intake × maternal atopy). None of the results obtained with models stratified on maternal atopy were significant.

Separate analyses were conducted for intake in the year before pregnancy and intake during the last trimester of pregnancy.

All analyses were performed using SAS version 9.3 (SAS Institute Inc, Cary, NC, USA). All P-values < 0.05 were considered statistically significant.

## Results

### Characteristics of the population

Table 2 presents the characteristics of our population of women and their newborns (*n* = 1140) and those of the entire cohort (*n* = 2002). Our population did not differ significantly from other the entire cohort with respect to all characteristics, except for the city of residence, gestational smoking, education level, income and percentage of women aged under 25 (Table 2). No woman in our population reported type 2 diabetes diagnosed prior to pregnancy or any chronic disease other than asthma, allergic rhinitis, atopic dermatitis and food allergies.

**Table 2:** Characteristics of mothers and newborns in the entire cohort (*n* = 2002) vs. the study population (*n* = 1140)

Variable	Mean ± SD or frequency (%)		P-value^†^
	*n* = 2002	*n* = 1140	
Mothers			
Center, %			
Poitiers	48.35	55.26	
Nancy	51.65	44.74	*0.0002*
Age (years) mean ± SD	29.99 ± 4.89	30.59 ± 4.71	0.10
< 25, %	15.08	11.07	
25–34, %	68.99	70.74	
> 34, %	15.93	18.19	*0.04*
BMI (kg/m^2^), mean ± SD	26.32 ± 4.46	26.28 ± 4.47	0.69
Normal, %	45.09	46.09	
Overweight, %	37.82	37.03	
Moderately obese, %	12.13	11.55	
Severely obese, %	4.96	5.33	0.94
Level of education, %			
Primary and below	7.56	4.54	
Secondary	61.05	59.34	
University and above	31.40	36.12	*0.005*
Household income, %			
> 3000 euros/month	25.82	29.47	*0.03*
Active smoking before pregnancy, %	36.72	30.93	*0.001*
Smoking during pregnancy (active + passive), %	31.92	22.54	*0.0001*
Maternal atopy	30.55	31.13	0.74
Supplementation with vitamins and/or trace minerals, %			
During the year preceding pregnancy only	5.87	5.87	
During the 3rd trimester of pregnancy only	49.70	49.10	
During both periods	21.50	22.20	
Never	22.94	22.83	0.98
Duration of gestation (WA), mean ± SD	39.22 ± 1.75	39.22 ± 1.75	0.97
Newborns			
Sex (girl), %	47.45	46.67	0.67
Birth weight (kg), mean ± SD	3.28 ± 0.59	3.29 ± 0.52	0.67
Premature, %^‡^	5.77	6.05	0.75
Number of older siblings, %			
0	48.60	48.25	
1–2	46.95	47.63	
≥ 3	4.45	4.12	0.88
Birth season, %			
Summer	26.58	26.14	
Autumn	22.22	22.54	
Winter	21.03	22.28	
Spring	30.17	29.04	0.82
Exclusive breastfeeding ≥ 4 months, %	13.39	15.09	0.20

*SD* standard deviation

^†^P-value: Chi squared test for categorical variables, Mann–Whitney U test for continuous variables; ^‡^ < 37 weeks of amenorrhea

### Health variables

By the age of 3, lifetime prevalence was of 10.18% for asthma, of 35.18% for wheezing, of 12.11% for allergic rhinitis and of 43.07% for atopic dermatitis.

#### Associations between maternal food intake before and during pregnancy and childhood asthma and allergies

Moderate (vs. low) intake of cooked green vegetables during the year before pregnancy was inversely associated with the risk of asthma by the age of 3 [OR (95% CI) = 0.60 (0.34–1.03), *P *= 0.06]. High preconceptional intake and moderate and high intakes of cooked green vegetables during the last 3 months of pregnancy had a non-significant tendency towards a negative relationship with the risk of asthma (Table 3).

**Table 3:** Adjusted associations between maternal diet before and during pregnancy and risk of wheezing in the child by the age of 3

Food category	During the year before pregnancy		During the last 3 months of pregnancy	
	OR (95% CI)^a^	P-value	OR (95% CI)^a^	P-value
Wheezing				
Meats				
Moderate vs. low	1.28 (0.92–1.78)	0.14	1.36 (0.99–1.88)	0.06
High vs. low	1.60 (1.15–2.22)	0.006	1.39 (1.01–1.92)	0.04
Asthma				
Cooked green vegetables				
Moderate vs. low	0.60 (0.34–1.03)	0.06	0.79 (0.45–1.36)	0.39
High vs. low	0.73 (0.43–1.26)	0.26	0.89 (0.51–1.57)	0.69
Allergic rhinitis				
Eggs				
Moderate vs. low	0.68 (0.44–1.04)	0.07	0.56 (0.33–0.93)	0.03
High vs. low	0.68 (0.42–1.09)	0.11	0.71 (0.43–1.18)	0.19
Raw vegetables				
Moderate vs. low	0.68 (0.43–1.07)	0.09	0.44 (0.26–0.74)	0.002
High vs. low	0.62 (0.40–0.98)	0.04	0.49 (0.29–0.82)	0.007
Grains				
Moderate vs. low	0.54 (0.33–0.88)	0.01	1.32 (0.80–2.18)	0.28
High vs. low	0.77 (0.49–1.22)	0.26	0.95 (0.55–1.63)	0.85
Cooked green vegetables				
Moderate vs. low	1.02 (0.64–1.63)	0.94	0.61 (0.37–1.00)	0.05
High vs. low	1.05 (0.65–1.69)	0.86	0.56 (0.33–0.97)	0.04
Meats				
Moderate vs. low	1.77 (1.09–2.90)	0.02	1.00 (0.60–1.67)	0.99
High vs. low	1.63 (1.00–2.67)	0.05	0.98 (0.50–1.42)	0.49
Atopic dermatitis				
Meats				
Moderate vs. low	1.02 (0.75–1.39)	0.90	0.90 (0.64–1.25)	0.53
High vs. low	1.33 (0.98–1.81)	0.07	1.09 (0.77–1.53)	0.64

*OR* odds ratio, *95% CI* 95% confidence interval

^a^Adjusted for maternal age, maternal pre-pregnancy mother’s BMI, mother’s active smoking before and during pregnancy, child’s exposure to environmental tobacco smoke (ages 0 to 3 years), humidity in the home (ages 0 to 3 years), maternal atopy, child’s sex, birth season, birth weight, exclusive breastfeeding ≥ 4 months, number of older siblings, mother’s education, household income, supplementation before and during pregnancy, and total daily energy intake (before or during pregnancy)

Moderate intake of eggs during pregnancy was protective against allergic [OR (95% CI) = 0.56 (0.33–0.93), *P *= 0.03)] Moderate intake of eggs before pregnancy was associated with a borderline significant reduced risk of allergic rhinitis [OR (95% CI) = 0.68 (0.44–1.04), *P *= 0.07]. Moderate and high intakes of raw vegetables during pregnancy were strongly associated with a reduced risk of allergic rhinitis (respectively, OR (95% CI) = 0.44 (0.26–0.74), *P *= 0.002 and OR (95% CI) = 0.49 (0.29–0.82), *P *= 0.007). Similarly, moderate and high gestational intakes of cooked green vegetables were associated with a reduced risk of allergic rhinitis [respectively, OR (95% CI) = 0.61 (0.37–1.00), *P *= 0.05 and OR (95% CI) = 0.56 (0.33–0.97), *P *= 0.04]. High intake of raw vegetables during the year before pregnancy was protective against allergic rhinitis [OR (95% CI) = 0.62 (0.40–0.98), *P *= 0.04]. Moderate intake of grains before pregnancy was inversely associated with the risk of allergic rhinitis [OR (95% CI) = 0.54 (0.33–0.88), *P *= 0.01]. Finally, significant associations were found between high preconceptional and gestational intakes of meats and the risk of wheezing [respectively, OR (95% CI) = 1.60 (1.15–2.22), *P *= 0.006 and OR (95% CI) = 1.39 (1.01–1.92), *P *= 0.04]. Moderate and high intakes of meats before pregnancy were also significantly associated with the risk of allergic rhinitis [respectively, OR (95% CI) = 1.77 (1.09–2.90), *P *= 0.02 and OR (95% CI) = 1.63 (1.00–2.67), *P *= 0.05]. A borderline significant association was found between high intake of meats before pregnancy and the risk of atopic dermatitis (Table 3).

## Discussion

In this prospective birth cohort study of French mother–child pairs, we found beneficial associations between cooked green vegetable consumption before pregnancy and childhood asthma; consumption of eggs and raw vegetables before and during pregnancy, consumption of grains before pregnancy, and consumption of cooked green vegetables during pregnancy and allergic rhinitis. To our knowledge, this study is the first to show a significant positive association between meat intake during the preconceptional period and a risk of wheezing, allergic rhinitis and atopic dermatitis in young children.

The beneficial effect of early regular consumption of cooked green vegetables, eggs, raw vegetables and grains before or during pregnancy on asthma, wheezing and allergic rhinitis in children may partly be due to the relatively high levels of antioxidants in these foods, but also to other nutrients found in these foods, notably vitamin D and n-3 (omega-3) polyunsaturated fatty acids (n-3 FA), which have been proven to have a protective effect against asthma and allergic diseases [30, 31]. Experimental models have shown that oxidant molecules can induce asthmatic reactions by provoking the release of proinflammatory mediators [32]. As a result, a low antioxidant diet may be associated to altered pulmonary development and consequently promote the development of wheezing and reduced respiratory function later in life. Certain antioxidant nutrients, such as vitamin E, flavonoids and selenium, in addition to their antioxidant properties, have immunomodulatory properties [33], which are protective against asthma and allergic diseases.

In addition, there is some evidence that early postnatal introduction of foods such as peanuts, fish and eggs might be beneficial in preventing allergies [34, 35]. More recently, a study found that a higher maternal intake of food allergens, including peanut and wheat, during early pregnancy was associated with a lower risk of allergy and asthma in the offspring at 8 years of age [36]. In our study, we showed that preconceptional and gestational exposure of potential allergens, including egg allergens, may also be beneficial for allergic rhinitis prevention, however results before pregnancy were borderline significant and only moderate intake of eggs during pregnancy may lower the risk of developing allergic rhinitis by the age of 3. In addition, we found that high consumption of eggs before pregnancy was inversely associated with the risk of wheezing, which may be an allergic disease symptom.

Regarding the associations found with meat intake, our results confirm findings of a previous study in children that showed a significant positive relationship between maternal meat intake during pregnancy and the prevalence of wheeze in the first year of life [9]. In addition, we found a significant association between high preconceptional intake of meats and risk of allergic rhinitis, which has not been demonstrated previously. One potential explanation of the association found with high meat intake is that some consituents of meat may affect the future development of symptoms such as wheezing and allergic diseases. Carcinogenic compounds, such as heterocyclic amines and polycyclic aromatic hydrocarbons are produced as meat is cooked at high temperatures [37]. In addition, meat intake results in exposure to* N*-nitroso compounds, which can form endogenously [38] and exogenously in nitrite-preserved meats [39]. As a result, these components found in meat may affect fetal immune system, although there is currently no epidemiological evidence of association between these carcinogens and wheeze and allergic diseases. Alternatively, meat intake might be an indicator of a specific maternal dietary pattern that may be related to an increased risk of wheeze and allergic diseases in children. Additionally, it may be possible that some unknown non-dietary factors related to meat intake may have confounded the observed associations.

Our results failed to reveal beneficial effects of certain categories of foods that have been demonstrated in previous studies to be protective against asthma and allergic diseases, notably fruit [40] and fish [40, 41]. One hypothesis that might explain this lack of association relates to recent and rapid adaptations by the agro-food system. With changes in lifestyles and technological and scientific advances, there has been a proliferation of new processed foods to respond to consumer demand. Many food products have become easier to store, transport, and conserve, designed to have a longer shelf life and to be prepared simply and quickly. However, this processing may have changed the nutritional content of certain foods. Some results suggest that the nutrient content of processed foods has changed, and may be lower in some vitamins and trace minerals [42]. Moreover, maternal fish consumption can be a source of fetal exposure to mercury [43] and other toxic substances [44]. The beneficial effect of fish might thus be offset by the presence of toxic contamination.

An original aspect of the present study is our examination of the relationship between childhood asthma and allergic diseases and maternal food intake during the year preceding the pregnancy. The significant associations that were found suggest that maternal nutritional factors before pregnancy may also play an important protective role against childhood asthma and allergic diseases. This observation raises the hypothesis that maternal diet during the periconceptual period, which is an important critical life stage, may have an impact on the epigenetic programming involved in the prevention (or early development, in the case of essential nutrient deficiencies) of asthma and allergic diseases. The nutrients in the foods that mothers consume before they become pregnant, at least during the year before pregnancy, may cause epigenetic changes and affect child health. One recent study showed that maternal supplementation with micronutrients during the periconceptual period was associated with changes in the epigenome of newborns [45]. Micronutrients appear to be involved in the methylation of some immune system genes. These epigenetic changes seem to be transmitted to the fetus during pregnancy, modifying genes involved in the development of the immune system, particularly in utero and in early childhood [45].

However, our study has some limitations. The use of FFQs to assess food intake can lead to errors as it is based on self-report and may be subject to memory bias, as well as misclassification bias (under-/over-estimation) when the women have difficulty assessing their intake, although if we adjusted for mother’s BMI. Moreover, as often in epidemiological study, we cannot conclude that there is a causal relationship between the consumption of certain food groups and child health. It could also be that lifestyle and personal health practices during pregnancy, factors that we were not able to assess, play an important role in the occurrence of allergic diseases. However, numerous results suggest that exposure to prenatal factors, including maternal diet, may have a greater influence on the development of asthma and of allergic diseases such as allergic rhinitis [46] by playing a role in the “programming” of fetal lung and immune development [47, 48]. In addition, the absence of significant associations between asthma and intake of food categories might be due to the low prevalence of this outcome, consequently limiting the power to detect a relationship between intake of food categories and the outcome. And finally, data on the children’s health were obtained through parental report via a questionnaire, which could also have led to misclassification bias. However, these questionnaires were based on the validated ISAAC questionnaires, decreasing the risk of such biases.

## Conclusions

The present study highlighted associations between maternal food intake before and during pregnancy and asthma and some allergic diseases in childhood.

Two public health interventions that can contribute to preventing asthma and allergic diseases in childhood are to provide women desiring pregnancy and pregnant women with recommendations on diet, notably concerning products with an effect on the development of allergic diseases and to promote healthy lifestyles. However, understanding the mechanisms by which dietary compounds promote or inhibit the development of allergies is essential to suggest specific and personalized guidelines for food avoidance or intake. Especially, a better understanding of the mechanisms relating metabolic and allergic diseases is crucial.

## Data Availability

The datasets used and/or analysed during the current study are available from the corresponding author on reasonable request.

## Abbreviations

EDEN: Etude des Déterminants pré et post natals du développement et de la santé de l′Enfant
ISAAC: International Study of Asthma and Allergies in Childhood
OR: odds ratio
CI: confidence interval
SD: standard deviation
